# Use of antimicrobials and antimicrobial resistance in Nepal: a nationwide survey

**DOI:** 10.1038/s41598-021-90812-4

**Published:** 2021-06-02

**Authors:** Komal Raj Rijal, Megha Raj Banjara, Binod Dhungel, Samarpan Kafle, Kedar Gautam, Bindu Ghimire, Prabina Ghimire, Samriddh Dhungel, Nabaraj Adhikari, Upendra Thapa Shrestha, Dev Ram Sunuwar, Bipin Adhikari, Prakash Ghimire

**Affiliations:** 1 Central Department of Microbiology, Tribhuvan University, Kirtipur, Kathmandu, Nepal; 2 Nepal Medical College, Jorpati, Kathmandu, Nepal; 3 Department of Public Health, Asian College for Advance Studies, Purbanchal University, Lalitpur, Nepal; 4 Mahidol-Oxford Tropical Medicine Research Unit, Faculty of Tropical Medicine, Mahidol University, Bangkok, Thailand; 5 Centre for Tropical Medicine and Global Health, Nuffield Department of Medicine, University of Oxford, Oxford, UK

**Keywords:** Antimicrobials, Policy and public health in microbiology, Microbiology

## Abstract

Nepal suffers from high burden of antimicrobial resistance (AMR) due to inappropriate use of antibiotics. The main objective of this study was to explore knowledge, attitude and practices of antibiotics uses among patients, healthcare workers, laboratories, drug sellers and farmers in eight districts of Nepal. A cross-sectional survey was conducted between April and July 2017. A total of 516 individuals participated in a face-to-face interview that included clinicians, private drug dispensers, patients, laboratories, public health centers/hospitals and, livestock and poultry farmers. Out of 516 respondents, 62.8% (324/516) were patients, 16.9% (87/516) were clinicians, 6.4% (33/516) were private drug dispensers. A significant proportion of patients (42.9%; 139/324) thought that fever could be treated with antibiotics. Majority (79%; 256/324) of the patients purchased antibiotics over the counter. The knowledge of antibiotics used among patients increased proportionately with the level of education: literate only [AOR = 1.4 (95% Cl = 0.6–4.4)], versus secondary education (8–10 grade) [AOR = 1.8 (95% Cl = 1.0–3.4)]. Adult patients were more aware of antibiotic resistance. Use of antibiotics over the counter was found high in this study. Knowledge, attitude and practice related to antibiotic among respondents showed significant gaps and need an urgent effort to mitigate such practice.

## Introduction

Over the last 50 years, antibiotics have been widely used in human and animal as prophylaxis, therapeutics, and growth promoters^1^. Nonetheless, widespread and inappropriate use of antibiotics has led to the emergence of antibiotic resistance—a condition in which pathogenic bacteria develop resistance to the specific antibiotic prescribed against it^2,3^. Emergence of drug resistance became even more prominent since the end of twentieth century when the declining efficacy of antibiotics were increasingly reported worldwide^4,5^. Increasing AMR constrains therapeutic options, prolongs hospital stays and mortalities, lowers quality of life, and adds to the economic burden^6^.

Antimicrobial resistance is a growing global health threat with multifaced phenomenon^7^. Infection with drug resistant microbes increases the morbidity, mortality, length of hospitalization and treatment cost of patients^7,8^. Such scenario will continue to affect low- and middle- income countries as they suffer from high overuse and misuse of antibiotics^9,10^. A multitude of factors contribute to the development of antimicrobial resistance. Increasing demands for food from animals and environment for a growing population has further added pressure in the eco-system. Also, increasing food and environmental links has facilitated the rapid transfer of drug-resistant pathogens^11^. In response to these challenges, a holistic ‘One Health’ approach has been advocated in recent years that aims to include the health of human, animal and the environment^12^. One health approach can be utilized to enhance risk analysis on emergence, spread and control strategies of AMR at the human-animal-local environment interfaces^13^.

A number of previous studies conducted in low- and lower middle- income countries (LMICs) have documented a poor level of knowledge, attitude and practice (KAP) on antibiotics prescription with remarkable over-the-counter (OTC) use which can ultimately lead to increase in AMR^10^. LMICs are adding more burdens to the ever-increasing AMR in comparison to the developed countries^14,15^. In LMICs, less than 40% of the patients attending public and private healthcare centers receive treatment according to the WHO guidelines^16^. Worryingly, healthcare professionals do not follow standard antimicrobial guidelines such as WHO guidelines and often prescribe broad spectrum antibiotics without laboratory evidence^17^.

Common to most LMICs, Nepal experiences an extremely huge burden of infectious diseases such as respiratory tract infections, enteric fever (typhoid, paratyphoid fever), urinary tract infections and other bacterial infections^9^. Researchers have reported high burden of drug resistant/multidrug resistant bacteria in Nepal^18–23^. Discrepancy between prescription and OTC use of antibiotic is found to contribute to the rise of AMR in Nepal^10^. Nonetheless, much less is explored around how behavior related to the use of antimicrobials in various sectors have contributed to the rising trend of AMR in Nepal^9,10^.

AMR surveillance in Nepal was commenced in 1999 by National Public health Laboratory (NPHL) with technical support from Bangladesh^24^. However, the AMR surveillance among animal pathogens started only in 2011 by joint efforts of NPHL and other veterinary laboratories^24^. Researchers have investigated different components of antimicrobial use and AMR in Nepal such as use of antibiotic in primary health care^25^, awareness of antibiotic use and its resistance in the general population^26,27^ and self-medication (with antibiotics) among medical students^28^. Nonetheless, these efforts have not been successful in documenting true burden of AMR in the country as these reports did not encompass all the sectors that used antimicrobials in various proportions. Traditionally, much of the focus has been invested in exploring the use of antimicrobials in humans, and use of antimicrobials among food animals is persistently neglected. To address these gaps in effective surveillance program, a Fleming fund has initiated a multi-disciplinary one health approach to tackle antimicrobial resistance in Nepal^29,30^. To the best of our knowledge, there are no KAP surveys on prescription and use of antibiotics through a comprehensive one health approach in Nepal. The main objective of this study was to explore knowledge and practices of antibiotics prescription and uses in various sectors utilizing a one health approach in eight districts of Nepal.

## Materials and methods

### Study settings

**Figure 1:**
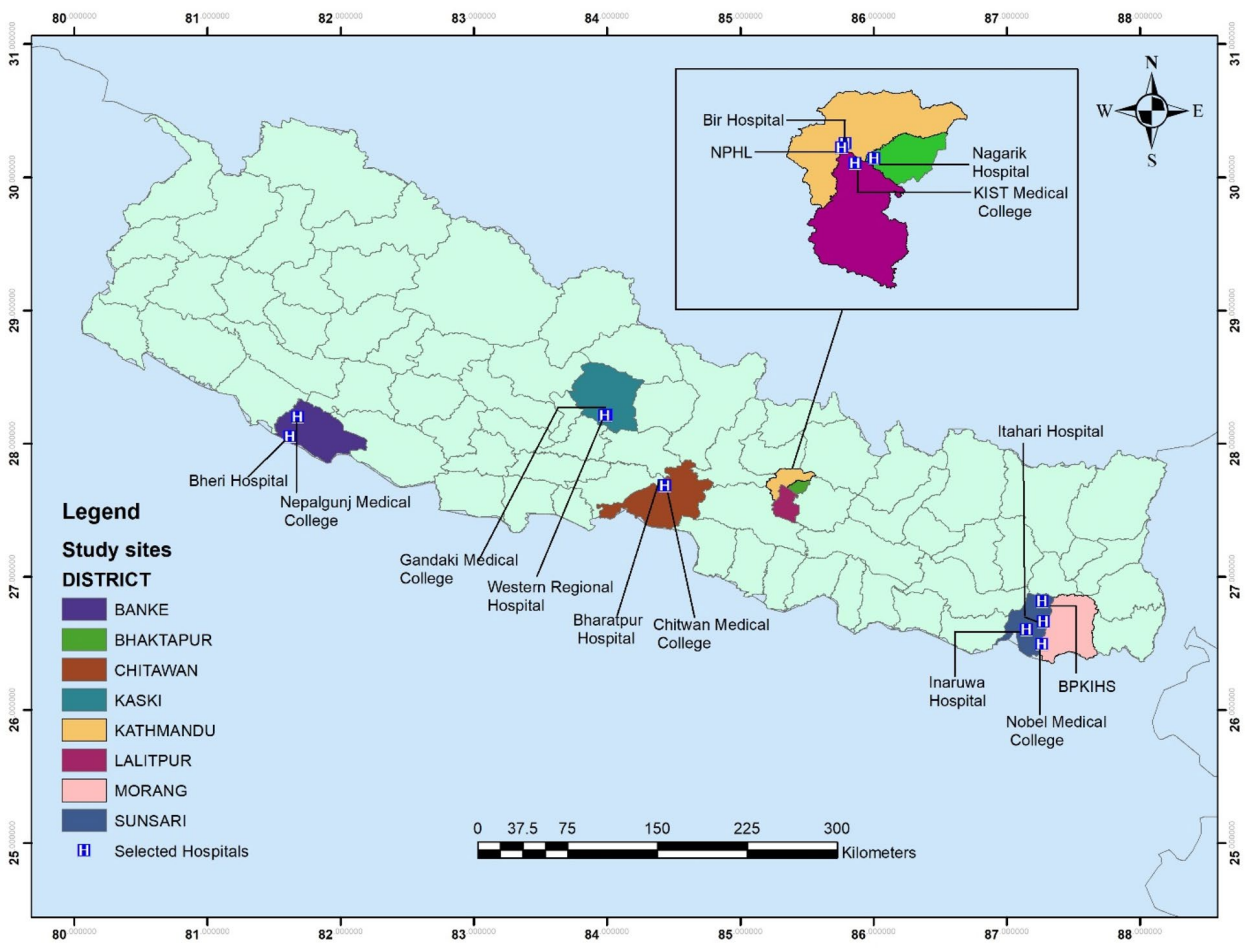
Study sites of selected eight districts of Nepal. The map was created using ArcGIS desktop version 10.8. The shapefile of the administrative districts and location for Nepal was obtained from the Government of Nepal, Ministry of Land Management, Survey Department website and were publicly available for unrestricted use (http://www.dos.gov.np/nepal-map)".

This study was conducted in selected eight districts of Nepal. Nepal is a landlocked mountainous country that shares 1800 km porous border with India in the east, west and south while it shares northern border with China^31,32^. It covers an area of 147,516 km^2^ between 26$\circ$22′–30$\circ$27′N and longitudes 80$\circ$04′–88$\circ$12′E. With varying population densities, the population of the country was 26.4 million in 2011^33^. Currently, Nepal is divided into seven provinces with 6 metropolitan cities, 11 sub-metropolitan cities, 276 municipalities, and 460 rural municipalities. The health system of Nepal is divided into three parts: Federal, Provincial and Local level health facility. In addition, private hospitals, private diagnostic centers, nursing homes, polyclinics and private drug dispensaries serve a large population in all seven provinces of Nepal. This study was conducted in tertiary hospitals and primary health care centers, private drug shops around the hospitals, laboratories (private/public), and food producing farmers and two rural primary health centers (PHC) from the selected eight districts of Nepal (Fig. 1). There are, a total of 4,863 public health care facilities in Nepal. The distribution of public health care facilities based on the provinces are: province 1 (n = 816), province 2 (n = 822), Bagmati province (province-3) (n = 934), Gandaki province (province 4) (n = 635), Lumbini province (province-5) (n = 741), Karnali province (province-6) (n = 404) and Sudurpashchim province (province-7) (n = 511) different health care facilities. In our study, 17 different health care facilities in seven different provinces were selected to represent the entire nation.

### Study population

Knowledge, Attitude and Practice (KAP) surveys were conducted among five groups (clinicians, patients, private drug sellers, livestock and poultry farmers and diagnostic laboratories) of key informants. A total of 550 participants were approached for the survey out of which (93.8%; 516/550) responses were received. 516 respondents in this study included clinicians and health workers (n = 87), private drug dispensers (n = 33), patients (n = 324), laboratory head/or representative of selected laboratories (n = 23), government hospital/ primary health centre (n = 17) and livestock farmers (n = 32).

### Study design

This was a descriptive cross-sectional survey using a previously validated and reliable WHO questionnaire between May 2017 and October 2017.

### Participant recruitment

This study was led by a research assistant in each province and supervised by co-investigator/principal investigator. At first a national and sub-national list of hospitals, pharmacies, and farms were obtained. Using a proportional sampling method, a proportional sample size for each category was calculated. Based on the sample size required for each category (hospitals/health facilities, pharmacies and farms) respondents were chosen using a simple random sampling. At the hospitals, patients were first requested and explained about the study. Broadly, patients who were willing to participate regardless of their illnesses were included in the study. They were also introduced about the procedure, risks and benefits of the study. All patients who agreed to participate in the study were included. The procedure was repeated for other categorical respondents. A written informed consent or thumb print from each participant or their guardian in case of children below 16 years was obtained. Written informed consents were obtained from health care workers, private drug sellers, Laboratory personnel, hospital/PHC pharmacist and food producing farmers from the list prior to the interview.

### Questionnaire development and pre-testing

Questionnaires were used following the WHO guidelines, publications and consultation with experts. Four different questionnaires for medicine prescribers, drug dispensers, patients and farmers were developed. Developed questionnaires were pre-tested during the orientation of the research assistants. Knowledge survey among HCWs consisted of three sections: firstly, on causes of emergence of antibiotic resistance, secondly on their existing know-how on treatment and development of drug resistance, and lastly on the mechanisms of antibiotic resistance. Pre-testing was conducted in hospitals and few sites within Kathmandu valley which were precluded from the data for this study.

### Methods of data collection

Six research assistants having a qualification of Masters of Science (M.Sc.) in Microbiology were recruited for this study. A week-long workshop was organized in Kathmandu to discuss about the study, protocol and pre-testing of the questionnaire. They were proportionately deployed in the study sites for data collection, enumeration and data entry. Principal and co-investigators provided the complete guidance during the entire study.

This study involved data collection at different levels—individual patients, healthcare providers, pharmacists, farms, and healthcare facilities. KAP on antimicrobial resistance were explored using standardized and pre-tested questionnaires adapted to each type of respondents^34^. Interviews were conducted with patients visiting health centers. All the participations were voluntary and were not provided any forms of incentives. The survey was conducted in collaboration with National Public Health Laboratory (NPHL) and the hospitals, data on AMR in different settings were collected from available reports which were tracked through laboratory registers/Laboratory Management Information System (LMIS) and were analyzed to assess AMR patterns. Assessment of recording, reporting and LMIS systems on antimicrobial resistance and review of the registers were also conducted in the laboratories. Information on morbidity and mortality related to AMR, through review of medical-records data were examined.

### Data management and analysis

All the questionnaires were screened for completeness, and errors; and were processed for data analysis. First, all the data were entered into Epi-data version 3.1 and were imported into SPSS software version 24.0 (IBM; Chicago, USA). Descriptive and univariate analysis was carried out using Chi-squared tests/Fisher’s exact test and are presented in supplementary files. Statistically significant variables based on the univariate analysis and research question were selected and were analyzed using multivariate logistic regression models. Various independent variables were controlled and evaluated to calculate adjusted odds ratio predicting the outcome variables that included knowledge of antibiotic and antibiotic resistance. A difference with p-value less than 0.05 was considered as statistically significant.

### Ethics approval and consent to participate

This study received ethical approval from Ethical review board (ERB) of Nepal Health Research Council (NHRC), (Reg No. 68/2017). Written informed consent was obtained from each participant for their voluntary participation in the study. This study was conducted in accordance with the Declaration of Helsinki.

## Results

### Demographic characteristics of respondents

Of the 87 health care workers, 75.9% (66/87) were male and 24.1% (21/87) were female. Majority of health care workers were medical doctors (90.2%;79/87). More than two-thirds of the health care workers belonged to age group 25–50 years (63.2%; 55/87), followed by age group (0–25) years (23%; 20/87) and > 50 years (13.8; 12/87). Of 324 patients interviewed, 72.8% (236/324 were male and 27.2% (88/324) were female. Half of the patients were from 26–50 years age group (50%; 162/324), followed by 0–25 years (32.7%;106/324) and > 50 years (17.3%; 56/324). Of 32 livestock farmers, 87.5% (28/32) were male and 12.5% (4/32) were female (Table 1). Their distribution in selected eight districts is summarized (Supplementary Table S1).

**Table 1:** Demographics characteristics of respondents.

Characteristics	Number (%)
**Health care workers (n = 87)**	
**Gender**	
Male	66 (75.9)
Female	21 (24.1)
**Age group (in years)**	
0–25	20 (23)
26–50	55 (63.2)
> 50	12 (13.8)
**Profession**	
Medical doctor	79 (90.2)
Paramedics	8 (9.8)
**Qualifications**	
MBBS only	42 (53.2)
MD General Practitioner	7 (8.9)
Internal Medicine	10 (12.7)
Pediatrics	2 (2.5)
Gynecology	3 (3.8)
Surgery	7 (8.9)
Others	8 (10.1)
**Working medical institutions**	
Public/ Government Hospital	37 (42.5)
Primary health care center	2 (2.3)
Private hospital of 100 beds or more	48 (55.2)
**Private Drug Seller (n = 33)**	
**Gender**	
Male	14 (42.4)
Female	19 (57.6)
**Age group (in years)**	
0–25	6 (18.2)
26–50	20 (60.6)
> 50	7 (21.2)
**Education**	
Pharmacy degree (D. Pharma, B. Pharma)	19 (57.6)
Health related other degree	10 (30.3)
Non-Health related degree	4 (12.1)
**Hospital Patients (n = 324)**	
**Gender**	
Male	236 (72.8)
Female	88 (27.2)
**Age group (in years)**	
0–25	106 (32.7)
26–50	162 (50)
> 50	56 (17.3)
**Type of patients**	
Inpatients	64 (19.8)
Out patients	260 (80.2)
**Education of the patients**	
Illiterate	40 (12.3)
Literate	36 (11.1)
Primary education (Class 1 -7)	36 (11.1)
Secondary Education (8–10)	97 (29.9)
Highest Secondary (10 + 2)	65 (20.1)
Bachelor and above	50 (15.4)
**Hospital /Primary Health Center, Chief / Representative (n = 17)**	
**Gender**	
Male	12 (70.6)
Female	5 (29.4)
**Age group (in years)**	
0–25	1 (5.9)
26–50	14 (82.4)
> 50	2 (11.7)
**Type of Health center**	
Primary health center	12 (70.5)
Hospital	5 (29.5)
**Livestock Farmers (n = 32)**	
**Gender**	
Male	28 (87.5)
Female	4 (12.5)
**Age group (in years)**	
0–25	4 (12.5)
26–50	20 (62.5)
> 50	8 (25)

### Knowledge

More than eighty percentage (range 80–96%) of the HCWs thought the role of various underlying factors (listed in questionnaire) on the emergence of resistance. Almost 40% of them were aware and had encountered different drug-resistant bacteria including extended spectrum beta-lactamase producing *Escherichia coli*, penicillin-resistant *Streptococcus pneumoniae* (PRSP), multi-drug resistant *Pseudomonas aeruginosa,* and Methicillin-resistant *Staphylococcus aureus* (MRSA). Of the listed mechanism of development of drug resistance in questionnaire, majority (69–91%) of the HCWs had sound knowledge (Supplementary Table S2).

Hospital patients were first asked about possibility of antibiotics in curing some bacterial and viral diseases. Nonetheless, in this study, we did not ask patients what they understood by the term ‘antibiotics.’ Out of 324 patients interviewed, 42.9% (139/324) of them thought that fever can be treated with antibiotics; more than one third (35.2%; 114/324) believed that antibiotics were used for treatment of cold and flu; 17.3% (56/324) believed that antibiotics can be used for treatment for sore throat, and 22.2% (72/ 324) believed that antibiotics cure headache and skin or wound infection. This study also assessed about their knowledge on terminologies related to resistance such as AMR, superbugs, drug-resistance, and drug-resistant bacteria. Of 324 patients, majority (84%; 272/324) of the respondents had never heard anything about antibiotic resistance and related terms and only 16% (52/324) of the patients had heard about different terms for antibiotics. Of the total (324) patients, 5.6% (18/324) heard about AMR and its related terms from media (newspaper, TV, radio); 3.7% (12/324) heard from family members and friend; 3.1% (10/324) heard from doctor or nurse; and a few (1.2%; 4/324) heard from pharmacists. Final section of the questionnaire explored on the perception on the drug resistance. Out of 324 patients interviewed, 75% of the respondents were not aware on anything related to antibiotic resistance issues (Supplementary Table S3).

Of 33 private drug sellers interviewed, majority (87.9%; 29/33) of them had heard about antibiotic resistance. However, a few (18%; 6/33) of them thought that antibiotics could treat all sorts of diseases listed in the questionnaire. Of 33 respondents, more than half (51.5%; 17/33) responded that antibiotics were used for treatment of bacterial diseases and oral thrush. Surprisingly, 23.3% (9/33) and 12.1% of them responded that antibiotics can be used to treat viral diseases and general weakness (Supplementary Table S4). Among livestock farmers, a few (12.5%; 4/32) of them had heard about antibiotic resistance. They had heard it mainly from the doctors or nurses (50%; 2/4), and pharmacists (50%; 2/4) (Supplementary Table S5).

### Attitude

Majority of HCWs (76/87) believed that antibiotics are overused all over the country; and majority (82/87) of them were in support of a more controlled policy for antibiotic use. Almost two-thirds of them (63.3%; 55/87) thought that AMR has already become significant problem in their hospitals. Out of 87 participants, majority (92%; 80/87) believed that prescribing broad spectrum antibiotics can aggravate the problem of resistance (Supplementary Table S6).

Most of the participants (92.6%; 300/324) believed that antibiotics should be used only when they are prescribed by a medical practitioner; exactly equal proportion of them also stressed on the fact that HCWs should prescribe antibiotics only when it is needed. Out of 324 patients, most (98.1%; 318/324) were well-informed on the hand hygiene practice. Nearly two-thirds of the participants (65.7%; 213/324) agreed that farmers should use antibiotics as minimum as possible. When they were asked on immunization, majority (96.6%;313/324) of the participants were certain that parents should ensure up to date vaccinations to their children. When it came to antibiotic resistance, they were unaware about this issue as 78.7% (255/340) of the respondents were either unaware or rejected the problem of AMR as one of the global crises. Nearly half of the respondents (161/324; 49.7%) were unsure about whether the AMR crisis has hit them individually. More than half (58.6%;190/324) were unsure on whether correct use of antibiotics could avoid AMR crisis at individual level. Another important finding was that 71.3% (231/324) of the individuals had no idea about their role in combating AMR crisis (Supplementary Table S7).

### Practice

**Figure 2:**
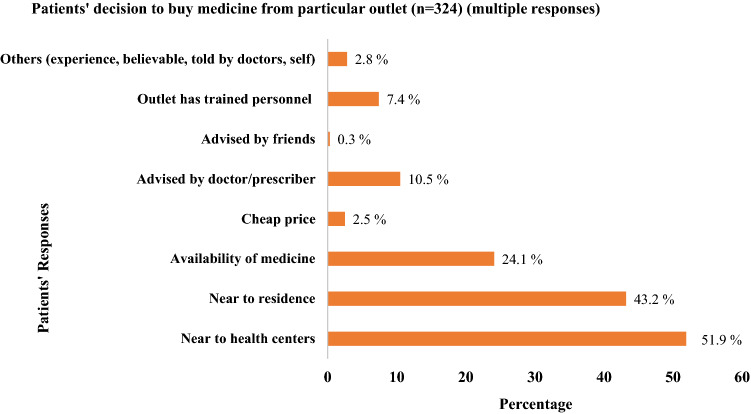
Patient’s responses to buy medicine from particular outlet.

Out of 87 HCWs, majority (71.3%; 62/87) reported that they prescribed antibiotics at least once a day, and only a few (13.8%; 12/87) prescribed at least once a week (Supplementary Table S8). Out of 324 patients interviewed, majority (79%; 256/324) of the respondents were purchasing antibiotics for themselves while 61.7% (200/324) respondents were currently taking antibiotics. Findings regarding the use of antibiotics among patients revealed that a few (18%; 58/324) of them used antibiotics without prescription (self-medicated); and few others (14.8%; 48/324) complained that they were not advised by HCWs on how to use the antibiotics. Almost half (46.3%; 150/324) admitted that they had the habit of discontinuing antibiotics (did not complete the prescribed dose) after they felt better (Supplementary Table S9). On patient’s response (multiple choice) to buy medicine from particular outlet, 51.9% (168/324) bought medicines from near the health centers and 43.2% (140/324) bought from near the residence (Fig. 2).

### Multivariate analysis

Multivariate analysis of people's knowledge on antibiotics showed that gender, age, person buying drug were not significant predictors. As the education level increased, the knowledge of patients on antibiotics also increased. Education level of patients was associated with the knowledge of antibiotics: literate only [ AOR = 1.4 (95% CI = 0.6–4.4)], primary education (1–7 grade [AOR = 1.0 (95% CI = 0.4–2.3)], secondary education (8–10 grade) [AOR = 1.8 (95% CI = 1.1–3.4)], Higher secondary (11–12 grade) [AOR = 1.6 (95% CI (0.8–3.1)] and bachelor and above [AOR = 2.0 (95% CI = 0.9–4.6)] (Table 2).

**Table 2:** Multivariate analysis of predictors of knowledge on antibiotics.

Variables	Regression coefficient	p-value	Odds ratio	95% CI for odds ratio	
				Lower	Upper
**Gender**					
Male	Ref	–	1	–	–
Female	0.093	0.723	1.097	0.657	1.833
**Age category (years)**					
< or 20	Ref				
21–40	0.108	0.715	1.115	0.622	1.997
41–60	0.175	0.612	1.191	0.606	2.344
> 60	0.438	0.294	1.550	0.683	3.514
**Education level**					
Illiterate	Ref		1		
Literate only	0.392	0.359	1.481	0.640	3.427
Primary education (1–7 grade)	0.073	0.856	1.075	0.491	2.357
Secondary education (8–10 grade)	0.627	0.042	1.872	1.024	3.422
Higher secondary (11–12 grade)	0.489	0.147	1.631	0.842	3.157
Bachelor and above	0.734	0.074	2.083	0.932	4.655
**Person buying the drug in the family**					
Self	Ref		1		
Other	−0.047	0.868	0.954	0.548	1.661

Related to knowledge about antibiotic resistance among patients, adults (41–60 years) were apparently more aware of antibiotic resistance. Knowledge of AMR on age group (21–40) years [AOR = 0.6 (95% CI = 0.3- 1.1)], age groups (41–60) years [AOR = 0.7 (95% CI = 0.3–1.5)].and > 60 years [AOR = 0.2 (95% CI = 0.07–0.73)]. Knowledge of antibiotic, person purchasing the drug were not associated with knowledge of antibiotic resistance; however, age of the patient was found as a predictor of knowledge of antibiotic resistance (Table 3).

**Table 3:** Multivariate analysis of predictors of Knowledge related to antibiotic resistance.

Variables	Regression coefficient	p-value	Odds ratio	95% CI for odds ratio	
				Lower	Upper
**Gender**					
Male	Ref		1		
Female	–0.401	0.186	0.670	0.370	1.213
**Age category (years)**		0.057			
< or 20	Ref		1		
21–40	–0.496	0.126	0.609	0.323	1.150
41–60	–0.324	0.399	0.723	0.341	1.535
> 60	–1.441	0.012	0.237	0.077	0.730
**Education level**		0.000			
Illiterate	Ref		1		
Literate only	–1.947	0.004	0.143	0.038	0.533
Primary education (1–7 grade)	–1.171	0.022	0.310	0.114	0.846
Secondary education (8–10 grade)	–0.736	0.036	0.479	0.240	0.953
Higher secondary (11–12 grade)	–0.296	0.421	0.744	0.362	1.529
Bachelor and above	1.017	0.019	2.766	1.180	6.484
**Person buying the drug in the family**					
Self	Ref		1		
Other	0.234	0.449	1.263	0.690	2.312
**Knowledge of antibiotic**					
No	Ref		1		
Yes	0.082	0.758	1.085	0.645	1.827

### The existing condition of LMIS on AMR in hospital/laboratories

**Figure 3:**
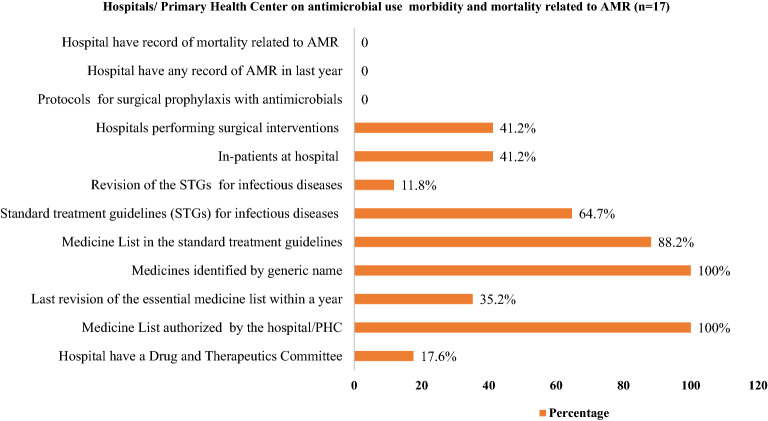
Hospitals provisions on antimicrobial use and morbidity and mortality related to AMR from Hospital/ PHC Record.

A total of 17 Hospitals/Primary health center were selected to gather information on antimicrobial use, morbidity and mortality related to AMR. Out of 17, all hospitals/primary health center had medicine by generic name and had an essential formulary list of medicines. Out of 17, only 35.2% had last revision of the essential medicine list with in one year and 17.6% had drugs and therapeutics committee. None of the hospital/PHC had protocols or norms for surgical prophylaxis, record of AMR in previous years and AMR related mortality (Fig. 3).

A total of twenty-three laboratories were assessed for performance, reporting and recording of AMR. More than half (13/23) of the laboratories were not purchasing ready-to-use culture media, thus were preparing those media manually as per routine requirements. In addition to this, 78.3% (18/23) of the laboratories did not have an automated blood culture system and relied on conventional procedures. Majority (69.5%; 16/23) of the laboratories did not have trends of performing biochemical assay for the confirmation of pathogenic organisms. Twenty laboratories were reliant on convention disk diffusion method (Kirby-Bauer method) for antimicrobial susceptibility testing (AST); only two laboratories adopted minimum inhibitory concentration (MIC) determination method for AST. However, majority (69.5%; 16/23) of the laboratories were following CLSI guidelines for interpretation of the result while all (100%; 23/23) of the labs did have AST recording system of the strains by clearly indicating them as resistant, intermediate or sensitive (Supplementary Table S10).

## Discussion

Rational use of antimicrobials is dependent on its main stakeholders that include patients, clinicians, veterinary professionals, drug retailers and farmers. It is essential to explore how these stakeholders interact, practice and use the antibiotics. A number of factors such as easy access to over the counter (OTC) use of drugs, lack of clear policy guiding empirical treatment regimens, poor literacy level among a major proportion of the population in the country, pressure among healthcare professionals for prescriptions, lack of proper diagnostic facilities, and other socioeconomic constrains were found to augment the irrational use of antimicrobial drugs^35^.

### Participants Knowledge on Antibiotics

Although this study did not explore what patients understood by the term ‘antibiotics’, majority of patients are not aware of what constitute such type of medicine, their scientific rationale and uses despite that ‘antibiotics’ were familiar term among the patients^35^. More than two-fifth of the patients still believed that fever was cured by antibiotics and echoed with previous studies from Nepal^27^ and Indonesia^36^. Around one-third of the respondents believed that viral diseases such as cold and flu, measles and HIV/AIDS could be treated with antibiotics. The widespread beliefs that antibiotics can treat a wide range of illnesses (ranging from non-specific body aches to the life-threatening viral diseases) may have multiple consequences including undermining the medical professional’s role in prescription and guidance^37^. In addition, beliefs that antibiotics could treat all diseases may have promoted buying of over-the-counter medications (self-medications), and also may have exerted pressure on HCWs to provide/prescribe antibiotics^9,10^.

In the context of basic knowledge on antibiotics, our study showed that the news media were reported to be the major source of information about the antibiotics in generating mass awareness^38^. A report published by the WHO also underscores the role of televisions, radios, internets, and press in spreading knowledge of antibiotics among general population^37^. Heightened awareness among participants regarding hand hygiene (98.1%) and immunization (96.6%) was observed in our study. The improved knowledge and practices may have been contributed by the mass media and vaccine campaign that served the population at the household over the last two decades^39^ and are reflected in previous studies from Nepal^40–45^.

### Knowledge on AMR

Overall, majority (84%) of the respondents had never heard about AMR while more than three-fourth of the total participants had no any idea on AMR related issues. This finding is similar (88.6%) to the previous study conducted in Nepal^46^. The poor knowledge on antibiotics may have impact on their attitude towards the use of antibiotics. Unlike their responses on knowledge questions, majority (93%) of the people stressed the need for the use of antibiotics by health professionals’ prescription only. When it came to the use of antibiotics, eighteen percentages of them were purchasing antibiotics over-the-counter (OTC) and were often driven by the distance, cost and perceived benefits. This finding is similar (24%) to one conducted in Bhutan^47^. Nonetheless, these responses suffer from social desirability bias (as patients very well knew that medicines ought to be taken with the prescription), and we believe OTC is way more prevalent than the reported percentage in this study. Even so, this trend highlights the urgent need for strict rules and regulations to discourage the OTC use of antibiotics^10^. Nearly half of the respondents (46.3%) in this study explained that they would not comply to the complete dose of medicines. Several previous studies have also concluded that the self-medication remains as one of the commonest practices in Nepal^48^. Most of the patients do not adhere to the treatment therapies as prescribed by the clinicians^49^ and thus do not follow a complete course of antibiotic therapy. Patients stop taking medicines once the disease symptoms begin to fade away and they stockpile the remaining drugs for future use. They often recommend such drugs to their family members, relatives and peers, which augments the rapid spread of the resistance^50,51^.

High prevalence of OTC medication practices could also be attributed to the low number of doctors to patients (1:1724) ratio in Nepal, which can inevitably oblige patients to rely on range and types of health workers available thus shaping the treatment seeking behavior^33,52^. Doctors are disproportionately distributed in Nepal. For instance, the Kathmandu valley has one doctor for 850 individuals but in rural zones the number is one doctor for each 150,000 individuals^53^. Moreover, Nepal is predominated by high proportion of rural and inaccessible areas where tertiary care centres are heavily constrained^54,55^. Therefore, the majority of the rural people rely on health assistants, quacks (unqualified persons pretending to have medical skills), and poorly trained drug dispensers for their treatment decision(s)^56^. Nonetheless, drug shops are a popular choice for OTC medication in both rural and urban regions and are affected by accessibility and functionality of the formal health services^35^. Despite that this study was conducted in a relatively urban areas, patients represented rural regions too. Follow-ups in patients from rural region were heavily constrained by their poor socio-economic conditions^57^.

### Factors influencing on KAP

Prescriber’s decisions may be affected by various factors such as lack of adequate professional knowledge on rational use of drugs, lucrative intensions often guided by themselves and/or pharmaceutical companies’ offer and/or hospital administration’s pressure, fear of losing clients (patients), patients’ self-demand, prescribing practices even for non-bacterial infections, and inappropriate doses and route of administration of the drugs^46^. Similarly, patients’ decisions on the use of antibiotics might be influenced by self-medication, sharing drug among family members, relatives and peers, ignorance of proper dose during medication, and lack of trust on medical professionals^58^. More importantly, poor infection control and augmented use of antibiotics in clinics, livestock and poultry farming, agriculture and aquaculture have aggravated the onset and rapid transfer of AMR globally^58^.

Education level of the respondents affected the knowledge and attitude related to antibiotics and AMR. Previous studies from Nepal^27^, India^59^, Bhutan^47^, Saudi Arabia^60^, Hong Kong^61^, Malaysia^62^, South Korea^63^, Oman^64^, Greece^65^, and Lithuania^66^ have echoed with our findings on the role of education. With higher education, respondents perhaps had good knowledge about proper use of antibiotics and adversities such as antibiotic resistance. As majority of them belonged to the age group of 25–50 years, their education level may have role rather than their age in affecting the KAP on antibiotics.

Healthcare workers, especially physicians are an integral stakeholder of antibiotics prescription and the AMR. Driven by the demand, and high prevalence of infectious diseases, health care workers are also one of the main facilitators of irrational prescriber of antibiotics^10,49^. In our study, more than eighty percentage (80–96%) of the HCWs had the knowledge on antibiotics, and AMR, and mechanisms leading to the emergence of resistance. This finding was slightly higher than a previous finding in Nepal^67^, India^68^, and was similar to other previous studies conducted in Egypt^69^, Zambia^70^ and Pakistan^71^. Improving knowledge and practice related to antibiotics might be attributable to the improved education and mass awareness carried out by media, activists, government, and NGO(s)/INGOs. In addition to this, improved status of surveillance, enforcement of law and orders could be other factors to be considered.

The majority of HCWs agreed that there is an overuse of antibiotics all over the country and almost two-third (63.3%) of them believed that AMR has already become a challenge in their own hospital(s). Similar findings were reported in USA in which 88% and 72% of the physicians agreed that AMR was a challenge in general and in their hospital^72^. In Egypt, 94% and 80% of the clinicians believed the AMR as a problem in their country and in their hospital^69^. Similar finding was reported in Nepal in which 87% of the physicians believed that antibiotics were overused throughout the country^67^. A previous study conducted in Nepal has reported that almost half the doctors (50.2%) used to prescribe antibiotics more than once a day^67^.

Previous studies have well documented that some of the healthcare professionals unnecessarily prescribed broad-spectrum antibiotics^73^. Minor health problems such as colds, coughs and diarrhea can be conservatively managed (without antibiotics). Nonetheless, clinicians prescribe antibiotics to these non-specific illnesses^50^. In addition to this, incorrect dose(s) and misleading guidance are also frequently observed in practices^50^. Similarly, pharmaceutical companies visit clinicians with lucrative offers which adds pressure on antibiotics prescriptions^74^.

Unwarranted demands from the patients’ side for any specific drug is another leading factor for irrational prescriptions. Such practices are predominant in the low-and middle-income countries (LMICs)^71^. Above all, poor infection control practices in the LMICs are often neglected but significant factor in the rapid spread of drug-resistant pathogens^48^.

### Policy implications

Findings from this study bear policy implications, particularly related to high rate of antibiotic prescription, OTC use of drugs, physicians’ neglect on AST guided therapy, and lack of knowledge on regulating policies for use of antimicrobials^48^. Common to resource constrained settings, Nepal suffers from scarcity of laboratory infrastructure, particularly in rural regions, which can inevitably lead to high empirical treatment^10^. Government should aim to establish laboratories with well-resourced with equipment and trained lab personnel^48^.

Nepal’s unique geographical landscape, population and urban–rural distribution of health services further affects the treatment seeking behavior including the (over-)use of antibiotics^75,76^. Aligning with these reports, HCWs also suggested for the culturally and geographically tailored guidelines for antimicrobial use in addition to the national guideline^68^. In addition, Nepal also suffers from disproportionate physician to patients’ ratio and should be properly addressed^77^. For example, by establishing the health care structure in rural regions and incentivizing the health care workers to serve the rural regions in addition to other developmental works^77,78^. At the same time, patients in remote regions of Nepal with simple infectious conditions should not be restricted to utilize antibiotics where qualified health care workers including doctors and tertiary care centers can be days away^9^. In addition, counselling on rationale use of antibiotics, and its adversities should be essential elements of prescription by health care workers which can be a sustainable method to increase the public awareness^10^.

Patients’ pressure also plays crucial role in the prescription and use of antibiotics which may be well reflected in the primary health centers where treatment options are limited and drugs are easily available OTC^10^. In such geographically remote regions, there is a good relationship between healthcare providers and community members, which creates pressure in prescription and sell of the antibiotics asked by patients rather than the appropriate ones^10,79–81^. Although policy guidelines demand for use of antibiotics based on the identification of causative agents, in practice, the empirical and over the counter use of antibiotics are high^9,10^.

As the positive roles of mass awareness and media are reported from several studies, promotion and incorporation of effective media/programs and routine trainings to HCWs are highly recommended. The importance of education and mass awareness in promoting the rational use of antibiotics are also supported by several previous studies in Nepal^82,83^ in India^83^ and in Europe^84^.

Antibiotic stewardships aligning with the ‘One Health’ approach need to be considered in future efforts in Nepal^85^. Lack of protocols for surgical prophylaxis, and record of AMR and associated mortality in this study implies need for investment in protocol development and robust surveillance in all the health care institutions. National and sub-national AMR surveillance agencies, hospitals, organizations and all stakeholders working in the sector of human health, livestock and agriculture can utilize the findings of this study to forge future strategies against AMR in Nepal. Collaborative efforts between ministry of health, ministry of agriculture & livestock development, WHO and their stakeholders are essential to implement ‘one health approach’. Government of Nepal (GoN) together with the leadership of WHO can forge ‘one health approach’ in national action plan on AMR.

## Strengths and limitations

Although this study explored knowledge, attitude and practice related to antibiotics uses among patients, healthcare workers, and farmers; using quantitative methods alone constrained us to understand the deeper reasons behind the responses including extent and nature on how they affect the use of antibiotics in Nepal. Future studies utilizing qualitative methods are critical to disentangle how these factors interact in various sectors to facilitate the use of antibiotics. For example, future ethnographic studies can explore why and how patients choose distance to the health center (against other factors) as an influence in buying antibiotics over the counter. This study was conducted in a select of districts/hospitals and inevitably constraints us to generalize the findings for the entire country. Although respondents in all categories were attempted to be balanced, number of pharmacists, and farmers were limited due to their proportion. In future, studies exploring farmers and pharmacists in greater proportion can help understand the true burden of antibiotic use in these sectors. Only 12.5% of farmers in our study had heard of AMR. This suggests need for more engagement with the animal and livestock sector in the future. Furthermore, this study did not include some of the underlying factors such as the extent of patients’ pressure in prescription, physician’s knowledge on microbiology laboratory reports, commercial interests in selling the antibiotics, pharmaceutical companies’ competing offers to clinicians. This survey may have incurred recall and social desirability bias. Data collected through prescription audit, observation and in-depth interviews were precluded in the analysis due to inconsistencies in their collection and inadequacy of the data which affected data saturation. Triangulation of various methods could have enriched our findings. Nonetheless, this study serves as a reference to the context and situation of AMR knowledge, attitudes and practices in Nepal.

## Conclusion

Although the level of knowledge, attitude and practice related to prescriptions and use of antibiotics apparently seems improved compared to previous studies, the prescription and overuse of antibiotics are unregulated in the study areas. Antibiotic stewardship programs in Nepal can at first address these gaps through health education, awareness and mass campaigns to mitigate the inappropriate use of antibiotics. Importantly, reformation in policy related to use of antibiotics should consider the multiple stakeholders that shape demand, supply, and compliance, including a ‘One Health approach’ to ensure animal health considerations are also taken into account.

## Supplementary Information

Supplementary Information 1.

Supplementary Information 2.

## Data Availability

The datasets used and/or analyzed during the current study are available from the corresponding author on reasonable request.

## Abbreviations

AMR: Antimicrobial resistance
ANM: Auxiliary Nurse Midwife
AST: Antibiotic Susceptibility Test
CLSI: Clinical Laboratory Standard Institute
EML: Essential Medicine List
ESBL: Extended Spectrum Beta Lactamase
HA: Health Assistant
HP: Health Post
KAP: Knowledge, Attitude and Practice
LMIS: Logistic Management Information System
MRSA: Methicillin Resistant *Staphylococcus aureus*
NPHL: National Public Health Laboratory
PHC: Primary Health Care Center
SPSS: Statistical Package for Social Sciences
WHO: World Health Organization
