# Supplementary material for: Use of antimicrobials and antimicrobial resistance in Nepal: a nationwide survey
Source: Sci Rep. 2021 Jun 2;11:11554. doi: 10.1038/s41598-021-90812-4 (PMC8172831; doi:10.1038/s41598-021-90812-4)
Supplement: Supplementary file 1 — Supplementary Information 1. [file 41598_2021_90812_MOESM1_ESM.docx]

**Supplementary Tables S1 to S10**

**Supplementary Table S1: Distribution of Respondents in different districts of Nepal**

| **Study District** | **Respondents** | | | | |  |
| --- | --- | --- | --- | --- | --- | --- |
|  | Health Care workers (n=87) | Private drug Seller  (n=33) | Hospital / PHC Pharmacy (n=17) | Patients (n=324) | Hospital/ Diagnostic Laboratories (n=23) | Farmers (n=32) |
|  | N (%) | N (%) | N (%) | N (%) | N (%) | N (%) |
| Morang | 12 (13.8) | 5 (15.2) | 2 (11.8) | 49 (15.1) | 3 (13) | 4 (12.5) |
| Sunsari | 12 (13.8) | 5 (15.2) | 1 (5.8) | 38 (11.7) | 3 (13) | 4 (12.5) |
| Chitwan | 12 (13.8) | 3 (9.05) | 3 (17.6) | 44 (13.6) | 3 (13) | 5 (15.6) |
| Kathmandu | 10 (11.5) | 4 (12.1) | 2 (11.8) | 57 (17.6) | 2 (9) | 4 (12.5) |
| Lalitpur | 8 (9.2) | 3 (9.05) | 2 (11.8) | 30 (9.3) | 3 (13) | 4 (12.5) |
| Bhakatapur | 10 (11.5) | 4 (12.1) | 2 (11.8) | 25 (7.7) | 3 (13) | 3 (9.4) |
| Kaski | 8 (9.2) | 5 (15.2) | 3 (17.6) | 40 (12.3) | 3 (13) | 5 (15.6) |
| Banke | 15 (17.2) | 4 (12.1) | 2 (11.8) | 41 (12.7) | 3 (13) | 3 (9.4) |

**Supplementary Table S2: Knowledge of antibiotics, antibiotics resistance, mechanism of AMR among Health care workers (n=87)**

| **Can you cite one or more cause of the antibiotic resistance emergence** | **Response** | **Number** | **Percentage** |
| --- | --- | --- | --- |
| The unnecessary prescription of antibiotics for viral infections | Yes | 82 | 94.3 |
|  | No | 5 | 5.7 |
| Inappropriate empiric choice of antibiotics | Yes | 78 | 89.7 |
|  | No | 9 | 10.3 |
| Inappropriate duration of antibiotic therapy | Yes | 78 | 89.7 |
|  | No | 9 | 10.3 |
| Too frequent prescription of “*broad-spectrum antibiotics*” | Yes | 82 | 94.3 |
|  | No | 5 | 5.7 |
| Lack of facilities/means/skills for bacterial diagnosis | Yes | 79 | 90.8 |
|  | No | 8 | 9.2 |
| Lack of guidelines on antibiotic usage | Yes | 81 | 93.1 |
|  | No | 6 | 6.9 |
| Random mutations in microbes | Yes | 81 | 93.1 |
|  | No | 6 | 6.9 |
| Patient demands and expectations for antibiotics, self-medications | Yes | 82 | 94.3 |
|  | No | 5 | 5.7 |
| Use of antibiotics in the livestock industry | Yes | 70 | 80.5 |
|  | No | 17 | 19.5 |
| Role of pharmaceutical companies in advertising and promoting use of antibiotics | Yes | 76 | 87.4 |
|  | No | 11 | 12.6 |
| The inadequate use by the patient, not respecting either dosage or duration of the treatment | Yes | 83 | 95.4 |
|  | No | 4 | 4.6 |
| **Can you cite one or more resistant bacteria prevalent in your institution?** | | |  |
| Multi-resistant *Pseudomonas aeruginosa* | Yes | 46 | 52.9 |
|  | No | 41 | 47.1 |
| Multi-resistant *Acinetobacter* spp. | Yes | 42 | 48.3 |
|  | No | 45 | 51.7 |
| Extended spectrum beta-lactamase producing *Escherichia coli* (ESBL *E. coli*) | Yes | 49 | 56.3 |
|  | No | 38 | 43.7 |
| Extended spectrum beta-lactamase producing *Klebsiella pneumoniae* | Yes | 39 | 44.8 |
|  | No | 48 | 55.2 |
| Penicillin-resistant *Streptococcus pneumoniae* (PRSP) | Yes | 46 | 52.9 |
|  | No | 41 | 47.1 |
| Methicillin-resistant *Staphylococcus aureus* (MRSA) | Yes | 52 | 59.8 |
|  | No | 35 | 40.2 |
| Vancomycin resistant *Enterococcus* (VRE) | Yes | 29 | 33.3 |
|  | No | 58 | 66.7 |
| ***Can you cite one or more mechanisms of antimicrobial resistance*** | | |  |
| Efflux pump | Yes | 60 | 69 |
|  | No | 27 | 31 |
| Alteration of binding site | Yes | 74 | 85.1 |
|  | No | 13 | 14.9 |
| Thickening of the cell wall | Yes | 60 | 69 |
|  | No | 27 | 31 |
| Enzymatic process | Yes | 79 | 90.8 |
|  | No | 8 | 9.2 |
| Intrinsic resistance (not acquired) | Yes | 65 | 74.7 |
|  | No | 22 | 25.3 |

**Supplementary Table S3: Knowledge of antibiotics and AMR among patients enrolled under study (n=324)**

| Variables | Response | Number | Percentage |
| --- | --- | --- | --- |
| Do you think these conditions can be treated with antibiotics?  **(Multiple responses, please read the responses)** | a. HIV/AIDS | 14 | 4.3 |
|  | b. Gonorrhea | 25 | 7.7 |
|  | c. Bladder infection or urinary tract infection (UTI) | 72 | 22.2 |
|  | d. Diarrhoea | 87 | 26.9 |
|  | e. Cold and flu | 114 | 35.2 |
|  | f. Fever | 139 | 42.9 |
|  | g. Malaria | 52 | 16 |
|  | h. Measles | 41 | 12.7 |
|  | i. Skin or wound infection | 72 | 22.2 |
|  | j. Sore throat | 56 | 17.3 |
|  | k. Body aches | 59 | 18.2 |
|  | l. Headaches | 72 | 22.2 |
| **Have you heard of any of the following terms?** | |  |  |
| Antibiotic resistance | No | 284 | 87.7 |
|  | Yes | 40 | 12.3 |
| Superbugs | No | 318 | 98.1 |
|  | Yes | 6 | 1.9 |
| Antimicrobial resistance | No | 313 | 96.6 |
|  | Yes | 11 | 3.4 |
| AMR | No | 312 | 96.3 |
|  | Yes | 12 | 3.7 |
| Drug resistance | No | 283 | 87.3 |
|  | Yes | 41 | 12.7 |
| Antibiotic-resistant bacteria | No | 297 | 91.7 |
|  | Yes | 27 | 8.3 |
| Do not hear | No | 52 | 16 |
|  | Yes | 272 | 84 |
| If yes, where did you hear about the term? **(Multiple response)** | a. Doctor or nurse | 10 | 3.1 |
|  | b. Pharmacist | 4 | 1.2 |
|  | c. Family member or friend | 12 | 3.7 |
|  | d. Media (newspaper, TV, radio) | 18 | 5.6 |
|  | e. Specific campaign | 2 | 0.6 |
|  | f. Others | 10 | 3.1 |
|  | g. Can’t remember | 11 | 3.4 |
| **Please indicate whether you think the following statements are ‘true’ or ‘false’** | | | |
| Antibiotic resistance occurs when your body becomes resistant to antibiotics and they no longer work as well | True | 71 | 21.9 |
|  | False | 24 | 7.4 |
|  | Don’t Know | 229 | 70.7 |
| Many infections are becoming increasingly resistant to treatment by antibiotics | True | 67 | 20.7 |
|  | False | 17 | 5.2 |
|  | Don’t Know | 240 | 74.1 |
| If bacteria are resistant to antibiotics, it can be very difficult or impossible to treat the infections they cause | True | 49 | 15.1 |
|  | False | 30 | 9.3 |
|  | Don’t Know | 245 | 75.6 |
| Antibiotic resistance is an issue that could affect me or my family | True | 60 | 18.5 |
|  | False | 26 | 8 |
|  | Don’t Know | 238 | 73.5 |
| Antibiotic resistance is an issue in other countries but not here | True | 19 | 5.9 |
|  | False | 60 | 18.5 |
|  | Don’t Know | 245 | 75.6 |
| Antibiotic resistance is only a problem for people who take antibiotics regularly | True | 37 | 11.4 |
|  | False | 47 | 14.5 |
|  | Don’t Know | 240 | 74.1 |
| Bacteria which are resistant to antibiotics can be spread from person to person | True | 48 | 14.8 |
|  | False | 30 | 9.3 |
|  | Don’t Know | 246 | 75.9 |
| Antibiotic-resistant infections could make medical procedures like surgery, organ transplants and cancer treatment much more dangerous | True | 55 | 17 |
|  | False | 24 | 7.4 |
|  | Don’t Know | 246 | 75.9 |
| Do you think antibiotics are widely used in agriculture (including in food-producing animals) in Nepal? | No | 205 | 63.3 |
|  | Yes | 36 | 11.1 |
|  | Do not know | 83 | 25.6 |

**Supplementary Table S4: Education and KAP of Private Drug seller under Study districts (n=33)**

| **Drug Seller (N=33)** | Number | % |
| --- | --- | --- |
| **Education** |  |  |
| Pharmacy degree (Diploma, B. Pharma) | 22 | 66.7 |
| Health related other degree (HA, ANM, CMA, LA) | 10 | 30.3 |
| Non-Health related degree | 1 | 3 |
| **Heard about Antimicrobial Resistance (AMR)** |  |  |
| Yes | 29 | 87.9 |
| No | 4 | 12.1 |
| **Knowledge of symptoms/diseases that can be treated with antibiotics** | |  |
| Antibiotic is used for the treatment of following diseases |  |  |
| **Response** | Yes | % |
| All diseases | 6 | 18.2 |
| Headache | 2 | 6.1 |
| Bacterial Diseases | 17 | 51.5 |
| Viral diseases | 9 | 23.3 |
| Oral Thrush | 17 | 51.5 |
| Sexually Transmitted Diseases | 27 | 81.1 |
| General weakness | 4 | 12.1 |

**Supplementary Table S5: Knowledge Attitude Practice of antibiotics on Livestock and poultry farmers (n=32)**

| **Variables** | **Responses** | **Number** | **Percentage** |
| --- | --- | --- | --- |
| Education level of the respondent | Illiterate | 6 | 18.8 |
|  | Literate | 2 | 6.3 |
|  | Primary level (1-7 class) | 3 | 9.4 |
|  | Secondary level (8-10 class) | 11 | 34.4 |
|  | Higher secondary level (11-12 class) | 9 | 28.1 |
|  | University education (Bachelor and above) | 1 | 3.1 |
| What do you do when animals get sick?  **(Multiple response)** | Call a veterinary technician | 26 | 81.3 |
|  | Use local medication | 8 | 25 |
|  | Give antibiotic to sick animals | 1 | 3.1 |
|  | Wait for self-recovery | 4 | 12.5 |
| Do you treat your animals yourself? | No | 24 | 75 |
|  | Yes | 8 | 25 |
| If not, how do you get your animals treated?  **(Multiple response)** | Call a veterinary doctor | 14 | 43.8 |
|  | Call a veterinary Technician (JT/JTA) | 16 | 50 |
|  | Call a quack | 6 | 18.8 |
| Do you know the use of antibiotic in treatment of animals? | No | 0 |  |
|  | Yes | 15 | 46.9 |
|  | Little information | 11 | 34.4 |
|  | Don't know | 6 | 18.8 |
| Do you use antibiotic yourself or recommended from somebody else?  **(Multiple response)** | Use myself | 5 | 15.6 |
|  | Recommended by veterinary technician | 25 | 78.1 |
|  | Recommended by drug seller | 14 | 43.8 |
|  | Recommended by neighbors / friends | 1 | 3.1 |
| Who prescribed antibiotic in your farm?  **(Multiple response)** | Veterinary doctor | 16 | 50 |
|  | Veterinary technician | 16 | 50 |
|  | Drug seller | 13 | 40.6 |
|  | Neighbors | 2 | 6.3 |
|  | Friends | 2 | 6.3 |
| Do you consume the milk of infected cattle/buffalo with mastitis? (n=21) | No | 9 | 42.9 |
|  | Yes | 12 | 57.1 |
| Do you sell the milk of the sick animals undergoing treatment with antibiotic? (n=21) | No | 10 | 47.6 |
|  | Yes | 11 |  |
| If no, how long you don't sell the milk in the market? (n=10) | Upto 24 hrs | 1 | 10 |
|  | Upto 48 hours | 3 | 30 |
|  | Upto to 4 days | 2 | 20 |
|  | Upto one week | 4 | 40 |
| Do you consume the milk or meat of the animals undergoing treatment with antibiotic? | No | 12 | 37.5 |
|  | Yes | 20 | 62.5 |
| Do you sell or market the goat, sheep pig etc while undergoing treatment with antibiotic? (n=21) | No | 15 | 71.4 |
|  | Yes | 6 | 28.6 |
| Do you use the antibiotic in poultry on the first day of chick arrival? (n=17) | No | 7 | 41.2 |
|  | Yes | 10 | 58.8 |
| Do you complete the dose of antibiotic in your flock? (n=17) | No | 4 | 23.5 |
|  | Yes | 13 | 76.5 |
| Have you ever used antibiotic for animals yourself without other prescription? | No | 22 | 68.8 |
|  | Yes | 10 | 31.3 |
| Do you know about antibiotic resistance? | No | 28 | 87.5 |
|  | Yes | 4 | 12.5 |
| If yes, where did you hear about the term? (n=4)  **(Multiple response)** | Doctor or nurse | 2 | 50 |
|  | Pharmacist | 2 | 50 |
|  | Family member or friend | 2 | 50 |
|  | Media (newspaper, TV, radio) | 1 | 25 |

**Supplementary Table S6: Perceptions (Attitude) of doctors, health workers on antibiotics use and antibiotic resistance (n=87)**

| **Antibiotic Uses and Resistance** | **Responses** | | | | | |
| --- | --- | --- | --- | --- | --- | --- |
|  | **Strongly disagree**  **N (%)** | | **Disagree**  **N (%)** | **No opinion**  **N (%)** | **Agree**  **N (%)** | **Strongly agree**  **N (%)** |
| Antimicrobials are overused nationally | | 2 (2.3) | 1 (1.1) | 8 (9.2) | 30 (34.5) | 46 (52.9) |
| Antimicrobial resistance is a significant problem at your hospital | | - | 9 (10.3) | 23 (26.4) | 36 (41.4) | 19 (21.8) |
| Better use of antimicrobials will reduce problems with antimicrobial resistant organisms | | 4 (4.6) | - | 6 (6.9) | 34 (39.1) | 43 (49.4) |
| New antimicrobials will be developed in the future that will solve with the problem of “resistance” | | 20 (23.0) | 10 (11.5) | 24 (27.6) | 28 (32.2) | 5 (5.7) |
| Antibiotic dispensation should be more controlled | | 2 (2.3) | - | 3 (3.4) | 34 (39.1) | 48 (55.2) |
| Strong knowledge of antimicrobials is important in my professional career | | 1 (1.1) | 1 (1.1) | - | 21 (24.1) | 64 (73.6) |
| Health workers need more education on antimicrobial resistance | | - | 1 (1.1) | - | 26 (29.9) | 60 (69.0) |
| Health workers need more education on the appropriate use of antimicrobials | | - | 1 (1.1) | - | 26 (29.9) | 60 (69.0) |
| **Antibiotic Resistance** | |  |  |  |  |  |
| Antibiotic resistance occurs when bacteria become resistant to antibiotics and they no longer work as well | | - | 1 (1.1) | 3 (3.4) | 55 (63.3) | 28 (32.2) |
| Many infections are becoming increasingly resistant to treatment by antibiotics | | - | - | 1 (1.1) | 35 (40.3) | 51 (58.6) |
| If bacteria are resistant to antibiotics, it can be very difficult or impossible to treat the infections | | - | 8 (9.2) | 7 (8.0) | 50 (57.5) | 22(25.3) |
| Antibiotic resistance is a global issue | | - | 1 (1.1) | 4 (4.6) | 32 (36.8) | 50 (57.5) |
| Antibiotic resistance is an issue that could affect me or my family | | - | 6 (6.9) | 10 (11.5) | 25 (28.7) | 46 (52.9) |
| Antibiotic resistance is only a problem for people who take antibiotics regularly | | - | 68 (78.2) | 6 (6.9) | 8 (9.2) | 5 (5.7) |
| Bacteria which are resistant to antibiotics can spread from person to person | | - | 19 (21.8) | 14 (16.1) | 24 (267.6) | 30 (34.5) |
| Antibiotic-resistant infections could make medical procedures like surgery much more dangerous | | - | 11 (12.6) | 4 (4.6) | 60 (69) | 12 (13.8) |
| Better use of antimicrobials will reduce problems with antimicrobials resistant organisms | | - | 7 (8.0) | 2 (2.3) | 60 (69) | 18 (20.7) |
| Prescribing broad spectrum antimicrobials when equally effective narrow spectrum antimicrobials are available increases antimicrobial resistance | | - | 6 (6.9) | 1 (1.1) | 25 (28.7) | 55 (63.3) |
| New antimicrobials will be developed in the future that will solve with the problem of resistance | | - | 31 (35.6) | 19 (21.9) | 32 (36.8) | 5 (5.7) |

**Supplementary Table S7: Perception of respondents (patients) to address the problem of antibiotic resistance (N=324)**

| **Statements** | **Responses** | | | | | |
| --- | --- | --- | --- | --- | --- | --- |
|  | **Strongly agree**  **N (%)** | | **Slightly agree**  **N (%)** | **Neither agree nor disagree**  **N (%)** | **Slightly disagree**  **N (%)** | **Strongly disagree**  **N (%)** |
| People should use antibiotics only when they are prescribed by a doctor or nurse | 165 (50.9) | | 135 (41.7) | 18 (5.6) | 6 (1.9) | 0 (0.0) |
| Farmers should give fewer antibiotics to livestock | 81 (25.0) | | 132 (40.7) | 90 (27.8) | 16 (4.9) | 5 (1.5) |
| People should not keep antibiotics and use them later for other illnesses | 90 (27.8) | | 103 (31.8) | 55 (17.0) | 69 (21.3) | 7 (2.2) |
| Parents should make sure all of their children’s vaccinations are up-to-date | 229 (70.7) | | 84 (25.9) | 11 (3.4) | - | - |
| People should wash their hands regularly | 294 (90.7) | | 24 (7.4) | 5 (1.5) | 1 (0.3) | - |
| Doctors should only prescribe antibiotics when they are needed | 185 (57.1) | | 115 (35.5) | 23 (7.1) | 1 (0.3) | - |
| Governments should reward the development of new antibiotics | 83 (25.6) | | 132 (40.7) | 99 (30.6) | 6 (1.9) | 4 (1.2) |
| Pharmaceutical companies should develop new antibiotics | 72 (22.2) | | 134 (41.4) | 109 (33.6) | 7 (2.2) | 2 (0.6) |
| Antibiotic resistance is one of the biggest problems the world faces | | 24 (7.4) | 45 (13.9) | 241 (74.4) | 12 (3.7) | 2 (0.6) |
| Medical experts will solve the problem of antibiotic resistance before it becomes too serious | | 12 (3.7) | 83 (25.6) | 215 (66.4) | 13 (4.0) | 1 (0.3) |
| Everyone needs to take responsibility for using antibiotics responsibly | | 79 (24.4) | 103 (31.8) | 140 (43.2) | 2 (0.6) | - |
| There is not much people like me can do to stop antibiotic resistance | | 16 (4.9) | 54 (16.7) | 231 (71.3) | 18 (5.6) | 5 (1.5) |
| I am worried about the impact that antibiotic resistance will have on my health, and that of my family | | 51 (15.7) | 104 (32.1) | 161 (49.7) | 7 (2.2) | 1 (0.3) |
| I am not at risk of getting an antibiotic-resistant infection, as long as I take my antibiotics correctly | | 38 (11.7) | 86 (26.5) | 190 (58.6) | 8 (2.5) | 2 (0.6) |

**Supplementary Table S8: Prescription Practice of antibiotics of Health Care workers (n=87)**

| **Character** | **Response** | **Number** | **Percentage** |
| --- | --- | --- | --- |
| How often to you prescribe antibiotics? | Rarely | 12 | 13.8 |
|  | At least once a month | 1 | 1.1 |
|  | At least once a week | 12 | 13.8 |
|  | At least once a day | 62 | 71.3 |
| A 28-year-old man is hospitalized with fevers, chills and productive cough. After a CXR, the diagnosis of community acquired pneumonia is made by a senior physician. IV levofloxacin is started. After 24 hours the patient feels better and his fever is improving, but he still has a productive cough. Blood and sputum cultures reveal *Streptococcus pneumoniae* (resistant to penicillin) and susceptible to fluoroquinolones. He is able to eat, oral absorption is good. | | | |
| **With regards to antimicrobial therapy, what would you do next?** | | | |
| ○ Continue intravenous levofloxacin | No | 54 | 62.1 |
|  | Yes | 33 | 37.9 |
| ○ Switch to oral levofloxacin | No | 38 | 43.7 |
|  | Yes | 49 | 56.3 |
| ○ Switch to ampicillin/ sulbactam | No | 79 | 90.8 |
|  | Yes | 8 | 9.2 |
| ○ Switch to piperacillin/ tazobactam | No | 80 | 92 |
|  | Yes | 7 | 8 |
| ○ Add vancomycin | No | 70 | 80.5 |
|  | Yes | 17 | 19.5 |
| ○ Switch to vancomycin plus piperacillin/tazobactam | No | 77 | 88.5 |
|  | Yes | 10 | 11.5 |
| A 50-year-old woman presents to clinic with 3 days of dysuria and feeling feverish. On exam she has a temperature of 101.8^o^ F, her blood pressure and heart rate are normal and at her baseline. There is mild costovertebral angle tenderness and suprapubic tenderness. She is menopausal but sexually active with only one male partner. Labs are ordered, including a urinalysis showing 80 white cells/high-powered field and large leukocyte esterase and nitrates; culture is pending. Renal function is normal. She has never taken antibiotics before and there is no documented antimicrobial resistance to urinary pathogens in this area. | | | |
| **What would you do next?** | |  |  |
| ○ Await culture results before starting an antimicrobial | No | 67 | 77 |
|  | Yes | 20 | 23 |
| ○ Treat with antibiotics immediately | No | 20 | 23 |
|  | Yes | 67 | 77 |
| ○ Mode of administration? | IV | 25 | 28.7 |
|  | IM | 22 | 25.3 |
|  | Oral | 40 | 46 |
| ○ Treat with antibiotics immediately and adjust if the organism is resistant | No | 17 | 19.5 |
|  | Yes | 70 | 80.5 |
| ○ Mode of administration? | IV | 28 | 32.2 |
|  | IM | 30 | 34.5 |
|  | Oral | 27 | 31 |
|  | Others | 2 | 2.3 |
| Is prophylactic antibiotic therapy prescribed before surgery in your hospital? | Never | 2 | 2.3 |
|  | Sometimes | 40 | 45.9 |
|  | Always | 45 | 51.7 |

**Supplementary Table S9: Patients Practice on Antibiotic Use and AMR (n=324)**

| **Variables** | **Response** | **Number** | **Percentage** |
| --- | --- | --- | --- |
| Whom did you buy the drug for? | Myself | 256 | 79 |
|  | Somebody else | 68 | 21 |
| When did you last take antibiotics? | Currently taking | 200 | 61.7 |
|  | In the last month | 64 | 19.8 |
|  | In the last 6 months | 13 | 4 |
|  | In the last year | 38 | 11.7 |
|  | More than a year ago | 6 | 1.9 |
|  | Never | 0 | 0 |
|  | Can’t remember | 3 | 0.9 |
| On the last occasion, did you get the antibiotics (or a prescription for them) from a doctor, health assistant or nurse? | No | 58 | 17.9 |
|  | Yes | 261 | 80.6 |
|  | Can't remember | 5 | 1.5 |
| On that last occasion, did you get advice from a doctor, health assistant, nurse or pharmacist on how to take them? | No | 48 | 14.8 |
|  | Yes, I received advice | 272 | 84 |
|  | Can’t remember | 4 | 1.2 |
| On that last occasion, where did you get the antibiotics? | Medical store or pharmacy | 310 | 95.7 |
|  | Stall or hawker | 6 | 1.9 |
|  | Somewhere/someone else | 1 | 0.3 |
|  | Can’t remember | 7 | 2.2 |
| When do you stop taking antibiotics once you’ve begun treatment? | When you feel better | 150 | 46.3 |
|  | Taken all of the antibiotics as directed | 161 | 49.7 |
|  | Don’t know | 13 | 4 |
| **Do you think this statement is ‘true’ or ‘false’?** | |  |  |
| *“It’s okay to use antibiotics that were given to a friend or family member, as long as they were used to treat the same illness”* | False | 72 | 22.2 |
|  | True | 189 | 58.3 |
|  | Do not know | 63 | 19.4 |
| *“It’s okay to buy the same antibiotics, or request these from a doctor, if you’re sick and they helped you get better when you had the same symptoms before”* | False | 114 | 35.2 |
|  | True | 153 | 47.2 |
|  | Do not know | 57 | 17.6 |
| What symptoms lead you to buy medicines at this place? | Fever | 129 | 39.8 |
|  | Headache | 114 | 35.2 |
|  | Coughing | 75 | 23.1 |
|  | Stomachache | 63 | 19.4 |
|  | Itchy genitals | 12 | 3.7 |
| Did you buy all the drugs as prescribed/advised? | No | 34 | 10.5 |
|  | Yes | 290 | 89.5 |
| When buying drugs what determines the amount you buy?  **(Multiple response)** | The dosage prescribed | 285 | 88.0 |
|  | The amount of money I have | 97 | 29.9 |
|  | The amount advised by friends/relatives/neighbor | 6 | 1.9 |
| What would you do if you/a patient experiences any side effect from the drug you buy at the outlet? **(Multiple response)** | a.    Go back to drug outlet | 125 | 38.6 |
|  | b.   Go to prescriber | 176 | 54.3 |
|  | c.    Seek medical attention elsewhere | 55 | 17 |
|  | d.   Seek advice from friend/relative | 2 | 0.6 |
|  | e.    See the drug seller | 29 | 9 |
|  | f.     Complain to an authority organization | 3 | 0.9 |
|  | g.   Take legal action | 6 | 1.9 |
|  | h.   Don’t do anything | 12 | 3.7 |
|  | i.     Other | 19 | 5.9 |
| As a drug consumer what do you consider to be your basic rights?  **(Multiple response)** | a.    Right to know the dose of the drug | 220 | 67.9 |
|  | b.   Right to know how to use the drug | 265 | 81.8 |
|  | c.    To know the side effects of the drug | 140 | 43.2 |
|  | d.   To know the price of drugs before buying | 77 | 23.8 |
|  | e.    To see the drug seller in case anything bad happens | 69 | 21.3 |
|  | f.     To return brought drugs | 86 | 26.5 |
|  | g.   To return bought drugs and get the refund | 85 | 26.2 |
|  | h.   Others | 11 | 3.4 |
|  | i.     Do not know | 11 | 3.4 |
| What information do you expect to get from a drug store? | a. Information on general health | 49 | 15.1 |
|  | b. Information on drugs | 212 | 65.4 |
|  | c. Information on diseases | 58 | 17.9 |
|  | d. Information in taking drugs | 73 | 22.5 |
|  | e. How to prevent disease | 25 | 7.7 |
|  | f. Side effects | 2 | 0.6 |
|  | g. Other | 3 | 0.9 |
|  | h. Do not know | 2 | 0.6 |

**Supplementary Table S10: Assessment of the recording, reporting and LMIS systems on antimicrobial resistance (n=23)**

| **Variables** | **Number** | **%** |
| --- | --- | --- |
| Purchase of ready to use culture media | 10 | 43.5 |
| Media quality control for growth and sterility | 14 | 60.9 |
| Use of biochemical assay for bacterial identification | 16 | 59.6 |
| Perform manual blood culture | 16 | 69.6 |
| *Automated blood culture system* |  |  |
| Yes | 5 | 21.7 |
| No | 18 | 78.3 |
| *Types of automated blood culture system* |  |  |
| BACTEC system | 4 | 80 |
| Biomerieux BACTALERT | 1 | 20 |
| Laboratory isolate fastidious bacteria |  |  |
| *H. influenzae, N. gonorrhea, N. meningitidis* | 10 | 43.6 |
| *Streptococcus pneumoniae* | 15 | 65.2 |
| **Antibiotic susceptibility tests (multiple responses)** |  |  |
| *Standard followed for AST* |  |  |
| CLSI | 16 | 69.5 |
| EUCAST | 2 | 8.7 |
| OIE | 1 | 4.3 |
| National standard | 4 | 17.4 |
| **Antibiotic susceptibility testing methods** |  |  |
| MIC determination | 2 | 8.7 |
| Gradient strip | 0 | 0 |
| Agar or broth dilution susceptibility | 1 | 4.3 |
| Disk diffusion (Kirby-Bauer method) | 20 | 86.9 |
| **Methods of conducting and interpreting antibiograms** |  |  |
| Manual (ruler sliding clipper) | 19 | 82.6 |
| Visual reading | 4 | 17.3 |
| **Laboratories using disk dispensers** |  |  |
| Yes | 8 | 34.8 |
| No | 15 | 65.2 |
| **Laboratory performing internal quality assurance on AST** |  |  |
| Yes | 13 | 56.5 |
| No | 10 | 43.5 |
| *Use of WHONET to record AST results* |  |  |
| Yes | 2 | 8.7 |
| No | 21 | 91.3 |
| **Storage practices of bacterial isolates in the hospital laboratories** | |  |
| Storage of bacterial isolates | 8 | 34.8 |
| Storage in liquid nitrogen | 1 | 12.5 |
| Storage in agar conservation stabs | 6 | 75 |
| Quality control and EQA, isolates are sent to confirmation/further analysis elsewhere | 6 | 26.1 |
| Laboratories have a clinical microbiologist | 9 | 39.1 |
| Laboratories test reference strains for AST | 10 |  |
| Daily | 1 | 10 |
| Monthly | 3 | 30 |
| At every change of disks or media | 6 | 60 |
| **Record AST test results and observations** |  |  |
| Laboratory notebook/paper based | 11 | 47.8 |
| Log book | 10 | 43.5 |
| Electronically | 12 | 52.2 |
| Result format for AST (resistant, intermediate, sensitive) | 23 | 100 |
| **Communication of test results** |  |  |
| To the clinician | 23 | 100 |
| To the public health office | 2 | 8.7 |
| To NPHL | 1 | 4.3 |
| *Submitted daily/monthly results to a National Reference Laboratory (NPHL)* | 8 | 34.8 |
