# Supplementary material for: Use of antimicrobials and antimicrobial resistance in Nepal: a nationwide survey
Source: Sci Rep. 2021 Jun 2;11:11554. doi: 10.1038/s41598-021-90812-4 (PMC8172831; doi:10.1038/s41598-021-90812-4)
Supplement: Supplementary file 2 — Supplementary Information 2. [file 41598_2021_90812_MOESM2_ESM.docx]

**Use of antimicrobials and antimicrobial resistance in Nepal: A nationwide survey**

Komal Raj Rijal^1*†^, Megha Raj Banjara^1†^, Binod Dhungel^1^, Samarpan Kafle^1^, Kedar Gautam^1^, Bindu Ghimire^1^, Prabina Ghimire^2^, Samriddh Dhungel^2^, Nabaraj Adhikari^1^, Upendra Thapa Shrestha^1^, Dev Ram Sunuwar^3^, Bipin Adhikari^4,5^, Prakash Ghimire^1*^

^1^Central Department of Microbiology, Tribhuvan University, Kirtipur, Kathmandu, Nepal

^2^ Nepal Medical College, Jorpati, Kathmandu, Nepal

^3^Department of Public Health, Asian College for Advance Studies, Purbanchal University, Lalitpur, Nepal

^4^Centre for Tropical Medicine and Global Health, Nuffield Department of Medicine, University of Oxford, Oxford, United Kingdom.

^5^Mahidol-Oxford Tropical Medicine Research Unit, Faculty of Tropical Medicine, Mahidol University, Bangkok, Thailand

†**Equally contributed**

*Corresponding author: Dr. Komal Raj Rijal, Central Department of Microbiology, Tribhuvan University, Kirtipur, Kathmandu. Email: [rijalkomal@gmail.com](mailto:rijalkomal@gmail.com), [komal.rijal@cdmi.tu.edu.np](mailto:komal.rijal@cdmi.tu.edu.np)

*Corresponding author Prof. Dr. Prakash Ghimire, Central Department of Microbiology, Tribhuvan University, Kirtipur, Kathmandu. E-mail: [prakash.ghimire@cdmi.tu.edu.np](mailto:prakash.ghimire@cdmi.tu.edu.np).

**Supplementary Materials**

1. Supplementary Table S1: Distribution of Respondents in different districts of Nepal
2. Supplementary Table S2: Knowledge of antibiotics, antibiotics resistance, mechanism of AMR among Health care workers (n=87)
3. Supplementary Table S3: Knowledge of antibiotics and AMR among patients enrolled under study (n=324)
4. Supplementary Table S4: Education and KAP of Private Drug seller under Study districts (n=33)
5. Supplementary Table S5: Knowledge Attitude Practice of antibiotics on Livestock and poultry farmers (n=32)
6. Supplementary Table S6: Perceptions (Attitude) of doctors, health workers on antibiotics use and antibiotic resistance (n=87)
7. Supplementary Table S7: Perception of respondents (patients) to address the problem of antibiotic resistance (N=324)
8. Supplementary Table S8: Prescription Practice of antibiotics of Health Care workers (n=87)
9. Supplementary Table S9: Patients Practice on Antibiotic Use and AMR (n=324)
10. Supplementary Table S10: Assessment of the recording, reporting and LMIS systems on antimicrobial resistance (n=23)
